# Mechanochemical Synthesis and Characterization of CuInS_2_/ZnS Nanocrystals

**DOI:** 10.3390/molecules24061031

**Published:** 2019-03-15

**Authors:** Erika Dutková, Nina Daneu, Zdenka Lukáčová Bujňáková, Matej Baláž, Jaroslav Kováč, Jaroslav Kováč, Peter Baláž

**Affiliations:** 1 Institute of Geotechnics, Slovak Academy of Sciences, 04001 Košice, Slovakia; dutkova@saske.sk (E.D.); bujnakova@saske.sk (Z.L.B.); balazm@saske.sk (M.B.); 2 Advanced Materials Department, Jožef Stefan Institute, Ljubljana 1000, Slovenia; nina.daneu@ijs.si; 3 Institute of Electronics and Photonics, Slovak University of Technology, 81219 Bratislava, Slovakia; jaroslav.kovac@stuba.sk (J.K.); jaroslav_kovac@stuba.sk (J.K.J.)

**Keywords:** CuInS_2_/ZnS, nanocrystals, mechanochemical synthesis, structural properties, surface properties, optical properties

## Abstract

In this study, CuInS_2_/ZnS nanocrystals were synthesized by a two-step mechanochemical synthesis for the first time. In the first step, tetragonal CuInS_2_ was prepared from copper, indium and sulphur precursors. The obtained CuInS_2_ was further co-milled with zinc acetate dihydrate and sodium sulphide nonahydrate as precursors for cubic ZnS. Structural characterization of the CuInS_2_/ZnS nanocrystals was performed by X-ray diffraction analysis, Raman spectroscopy and transmission electron microscopy. Specific surface area of the product (86 m^2^/g) was measured by low-temperature nitrogen adsorption method and zeta potential of the particles dispersed in water was calculated from measurements of their electrophoretic mobility. Optical properties of the nanocrystals were determined using photoluminescence emission spectroscopy.

## 1. Introduction

Ternary metal chalcogenide nanocrystals have attracted considerable interest in the past decade because of their numerous applications in photoelectronic, thermoelectric devices and biotechnology [1,2,3,4,5]. Copper indium disulphide (CuInS_2_ (CIS)) is a ternary chalcogenide semiconductor from the chalcopyrite family, extensively studied due to its high absorption coefficient, suitable band gap, good radiation stability, easy conversion of n/p carrier type, low toxicity, large Stokes shifts, and high emission intensities [6,7]. Therefore, CIS is considered to be an alternative low-toxic material for bio-imaging and solid-state lighting, as well as suitable candidate for solar cell devices [8,9]. In the beginnings, CIS quantum dots (QDs) commonly showed luminescence with very broad photoluminescence spectra probably caused by polydisperse distribution of nanoparticles [10,11]. Recent works on CIS QDs have found procedures to increase quantum yields and photoluminescence intensities [12,13]. Moreover, their combination with another inorganic semiconductor with a wider band gap (ZnS) can lead to even better optical properties due to elimination of surface non-radiative recombination defects. Therefore, CuInS_2_/ZnS core-shell NCs may be used in solar cell structures as absorbing materials [14]. The structural and optical properties of CuInS_2_/ZnS QDs were investigated for application as light-emitting diodes [15,16] and it has been shown that large-scale synthesis of highly emissive and photostable nanocrystals is possible in hybrid flow reactor [17]. Magnetic CuInS_2_-ZnS nanocomposites for bioimaging were also prepared [18,19].

Several approaches for preparation of inorganic coated CIS nanomaterials have been explored, including solvothermal route [15], wet chemical procedure [18], precursor thermal-decomposition method [20], non-injection synthesis [21], colloidal synthesis [22], by heating up method [23].

In this paper, CuInS_2_/ZnS nanocrystals were prepared by dry high-energy milling. The structural, surface and optical properties of the sample were investigated. To our best knowledge, the CuInS_2_/ZnS nanocrystals prepared by mechanochemical synthesis were not reported until now. The novelty of this work is the simple mechanochemical preparation method of CuInS_2_/ZnS nanocrystals with interesting properties in a very short time, at ambient pressure and temperature. 

## 2. Results and Discussion

### 2.1. Why Mechanochemistry?

Mechanochemistry is a non-conventional method considered as one of the cost and time effective methods towards preparation of novel and high-performance nanomaterials. Today, mechanochemistry belongs to branches of chemistry with many applications. The top-down mechanochemical approach is also prospective preparation technique, because of many advantages related to the milling process. The simplification of the synthesis processes with their reproducibility and easy way of operation, ecological safety and the product extraordinariness (nanoscale aspects) emphasizes the suitability of mechanochemistry application. By mechanochemical synthesis it is possible to control and regulate the course of solid state reactions and phase transformations. The main advantage in comparison with traditional technological procedures is a decrease in the number of technological stages, excluding the operations that involve the use of solvents and gases and the possibility of obtaining a product in the metastable state which is difficult or impossible to obtain using traditional technological methods. The environmental aspects of these processes are particularly attractive [24,25,26]. Several chalcogenide/ZnS nanocrystals obtained by mechanochemical synthesis by our research group was published in several papers [27,28,29,30,31,32]. Regarding to the topic of this paper–preparation of CuInS_2_/ZnS, the core-shell structures or QDs have been predominantly prepared previously e.g. in [15,21,33,34]. Chuang et al. [15] prepared CuInS_2_/ZnS core/shell quantum dots (QDs) with varying [Cu]/[In] ratios using a step wise solvothermal route by heating of solutions at 200 °C for 14 h and using solvents. Chen et al. [33] also synthesized high-quality CIS/ZnS QDs at the gram scale by using CuI, In(OAc)_3_ and 1-dodecanethiol as precursors and subsequent application of ZnS shell coating and CuInS_2_–ZnS alloying. Zhang et al. [34] prepared non-blinking (Zn)CuInS/ZnSQDs in organic phase through in situ interfacial alloying approach in three complex steps, namely synthesis of CuInS QDs, eliminating the interior traps of QDs by forming graded (Zn)CuInS alloyed QDs, and modifying the surface traps of QDs by introducing ZnS shells onto (Zn)CuInS QDs using alkylthiols as sulfur source and surface ligands. Nam et al. [21] synthesized colloidal CIS core/shell QDs through a facile non-injection, one-pot approach by reacting Cu and In precursors with dodecanethiol dissolved in 1-octadecence at 220 °C. In all mentioned papers, the synthesis of CuInS_2_/ZnS core-shell QDs took place for a long time, at relatively high temperatures and pressures and moreover, by using solvents. On the contrary, in our case we prepared pure CuInS_2_/ZnS nanocrystals (with yield of product 97%) for only 30 min of the mechanochemical synthesis with out using some organic solvents, at ambient temperature and pressure. The solid state approach via mechanochemistry can be realized through simple solvent-free technology. Moreover, the mechanochemical approach is reproducible, ensuring high yield, easy to operate and very important point is scale up, as was shown in several latest papers describing the preparation of similar materials in semi-industrial scale [35,36,37]. In the next part, the structural, surface and optical properties of mechanochemically synthesized CuInS_2_/ZnS nanocrystals are described. However, in our case, we have not obtained typical core-shell structures. The reactions for the preparation of CuInS_2_/ZnS nanocrystals together with the milling conditions and overall flowsheet of the process are described in Materials and Method part.

### 2.2. Structural Characterization

**Figure 1:**
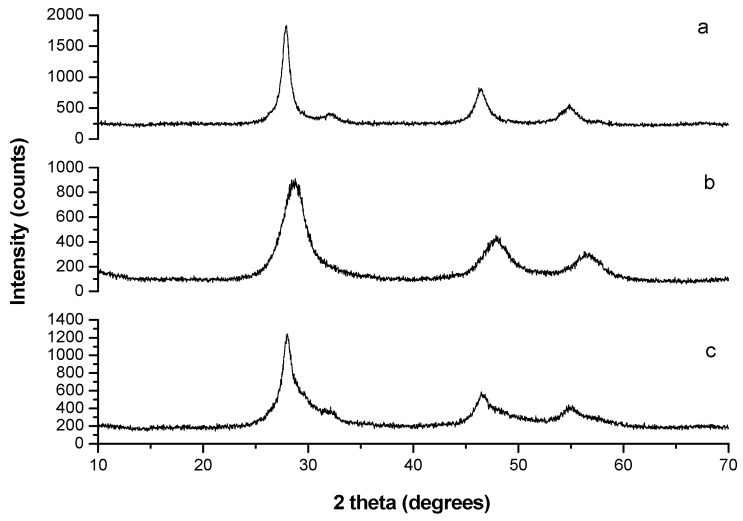
XRD patterns of (**a**) CuInS_2_; (**b**) ZnS and (**c**) CuInS_2_/ZnS nanocrystals.

The X-ray diffraction (XRD) results of mechanosynthesized CuInS_2_ (JCPDS-03-065-1572) and ZnS (JCPDS-01-072-9266) are shown in Figure 1a,b. The broad diffraction peaks were observed due to size of CuInS_2_ and ZnS crystallites in the nano range. Rietveld analysis was used for determination of the crystallite sizes and the obtained results were d = 8 ± 2 nm and d = 2.5 ± 0.5 nm for CuInS_2_ and ZnS phases, respectively. In the case of mechanochemically synthesized CuInS_2_/ZnS nanocrystals in molar ratio CuInS_2_:ZnS = 1:4, the XRD pattern shown in Figure 1c proves the coexistence of both components. All the peaks exhibited a slight shift toward higher angles, closer to the peak positions of bulk ZnS. This shift can be caused by the lattice mismatch between CuInS_2_ and ZnS nanoparticles. A similar behaviour was observed in other studies [16,21,38].

**Figure 2:**
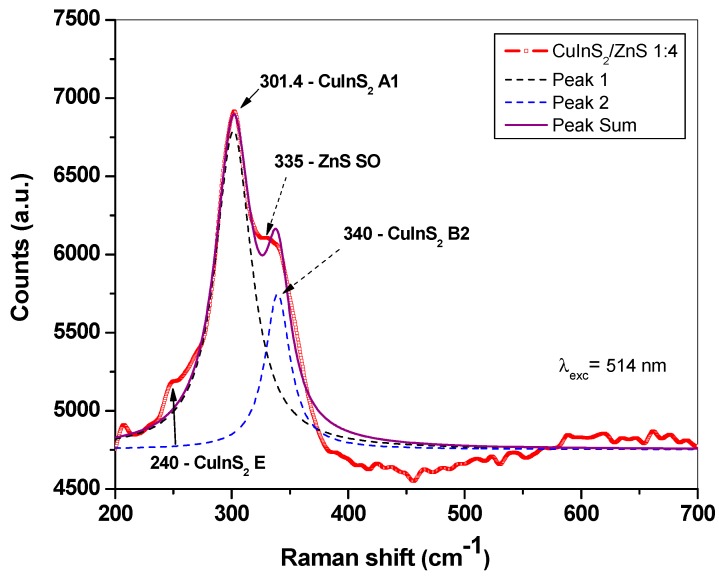
Raman spectrum of CuInS_2_/ZnS nanocrystals excited at 514 nm.

The Raman spectroscopy was used for the confirmation of phases identified by XRD. Raman spectrum from mechanochemically synthesized CuInS_2_/ZnS nanocrystals excited by Ar laser at 514 nm is shown in Figure 2. It can be seen that the dominant feature of the Raman plot is the peak at 301.4 cm^−1^ and the weaker peaks at 240 cm^−1^ and 340 cm^−1^ (not resolvable), which were previously assigned to the A1, E, and B2 modes of CuInS_2_ phase respectively [39,40]. The broader peak at 335–340 cm^−1^ can be assigned to mixed prevailing surface optical (SO) mode of ZnS (335 cm^−1^) and weak B2 mode of CuInS_2_ (340 cm^−1^). This is in accordance with the results observed from experiment, which has been discussed in a previous studies on ZnS nanowires, where SO phonon mode varies in wavenumber depending on the shape and surface roughness of the ZnS nanostructures [41,42].

**Figure 3:**
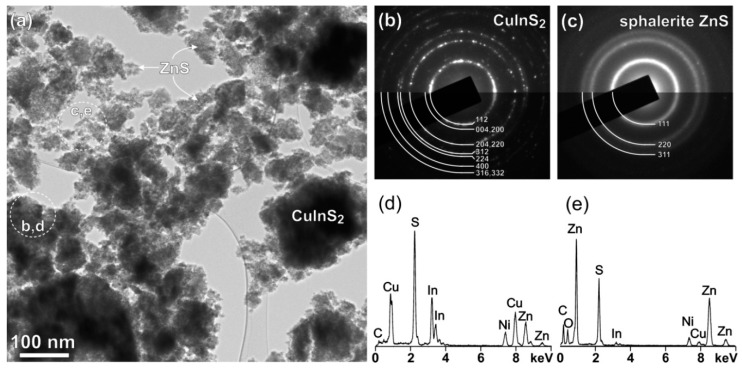
(**a**) Low-magnification image of the CuInS_2_/ZnS nanocrystals. SAD pattern and EDS spectrum taken from areas including mostly CuInS_2_ (**b**,**d**) and ZnS (**c**,**e**) nanoparticles.

The sample was further characterized by transmission electron microscopy (TEM) in detail. Low-magnification image of the sample (Figure 3a) shows that it is composed of partially agglomerated crystallites with sizes in the nanometer range. A more detailed analysis reveals that parts with darker and more uniform contrast belong to the areas with prevailing CuInS_2_ phase, while parts with grainy contrast are agglomerates of nanocrystalline ZnS. Areas containing mainly CuInS_2_ phase and ZnS were analyzed by SAD and EDS and the results are shown in Figure 3c,d, respectively. A comparison of SAD patterns taken from areas containing mainly CuInS_2_ and ZnS phases shows that both phases have different crystallite size. The SAD pattern taken from the CuInS_2_ phase is composed of dotted rings while rings with diffused contrast are characteristic for ZnS parts of the sample. This indicates that the size of the CuInS_2_ crystallites is larger than the size of the crystallites of ZnS. Diffraction rings originating from the area including mostly CuInS_2_ fit well to the roquesite phase, as indicated already by the XRD analyses (Figure 1). The three most intense rings from nanocrystalline ZnS fit best to the sphalerite ZnS, which is the stable low-temperature ZnS modification. EDS analyses were applied to confirm the local chemical composition of different phases in the sample. Spectra from the areas including CuInS_2_ phase always contain some Zn because ZnS nanoparticles or clusters of nanoparticles are always present nearby the surface of CuInS_2_ or in some cases covered by ZnS nanocrystals.

**Figure 4:**
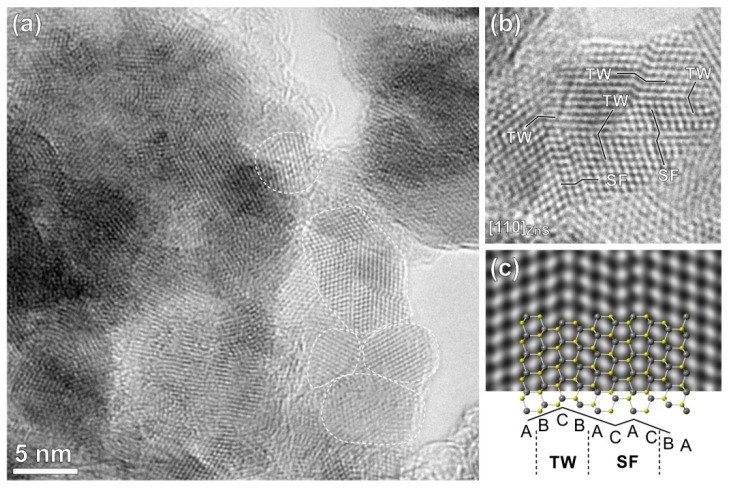
HRTEM image of (**a**) the ZnS nanoparticles revealing the size of the crystallites around or below 10 nm; (**b**) ZnS crystallite oriented along the (110) zone axis; (**c**) Wurtzite-type planar defects. The visible twin boundary and stacking fault defects are shown.

The ZnS nanoparticles were examined in more detail by high-resolution TEM (HRTEM). Figure 4a shows that the ZnS part of the sample is composed of tightly packed nanoparticles with average size around 10 nm or lower. HRTEM image of a ZnS crystallite oriented along the (110) zone axis (Figure 4b) confirms that the structure of ZnS is sphalerite with the cubic close packed (*ccp*) sulphur sublattice (-A-B-C- stacking of the sulphur planes along all 〈111〉 directions). Inside the cubic matrix, a high density of wurtzite-type planar defects with local hexagonal stacking (-A-B-A-) are observed. The defects are simple twin boundaries and stacking faults. Figure 4c shows a simulated image and atomic model of sphalerite ZnS with a twin boundary (-A-B-C-B-A-) and a stacking fault (-B-A-C-A-C-B-A-). Wurtzite-type planar defects are common in ZnS [43] and there are different reasons for their formation, like synthesis conditions close to the sphalerite-wurtzite phase transition temperature or the presence of dopants like copper and oxygen, which stabilize the local wurtzite stacking. In the present case, the wurtzite-type planar defects were probably introduced into the sphalerite ZnS by mechanical deformation during high-energy milling; however, stabilization of the local hexagonal stacking by oxygen is probable, since wurtzite is the stable modification of ZnO and already the presence of a small amount of oxygen (see EDS spectrum in Figure 4e) will favor formation of the local oxygen-rich hexagonal wurtzite-type stacking inside the cubic ZnS matrix.

### 2.3. Optical Properties

**Figure 5:**
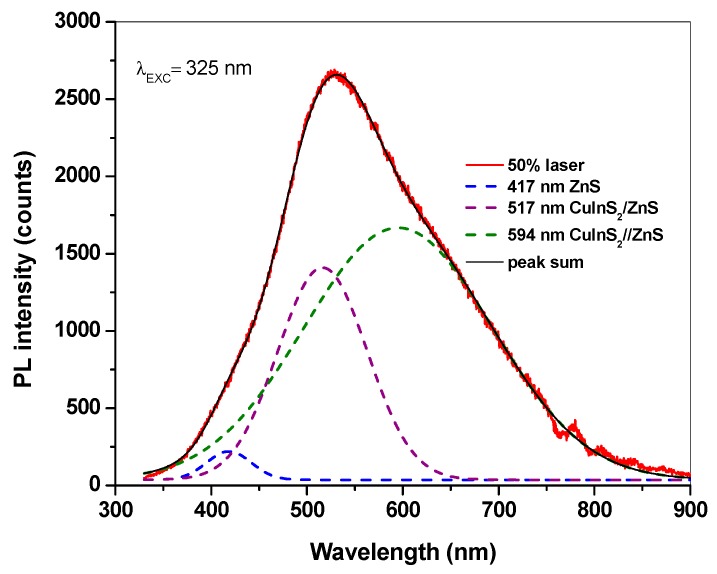
Micro-PL spectra of CuInS_2_/ZnS nanocrystals.

The optical properties of the mechanochemically synthesized CuInS_2_/ZnS nanocrystals were recorded by using micro-photoluminescence (micro-PL) spectroscopy. The room temperature micro-PL spectrum with the excitation wavelength at 325 nm is presented in Figure 5. The spectrum displayed a broad emission located between the peaks for bulk sphalerite ZnS and roquesite CuInS_2_. There are three resolvable peaks in the deconvoluted measured spectra. The emission peak located at 417 nm (2.96 eV—blue emission) can be attributed to ZnS and the peaks at 517 nm (2.38 eV—green emission) and 594 nm (2.08 eV—yellow emission) can be assigned to CuInS_2_/ZnS size dependent luminescence. The emission peaks are blue-shifted in relation to the band gap and may be attributed to band emissions from different small nanocrystalline domains or clusters and also to partial exchange between Zn and cations from CuInS_2_ (predominantly) with in [44]. The peak observed at 417 nm is in accordance with the weak one observed for ZnS published by Lee et al. in paper [45]. The peak at 517 and 594 nm may be attributed to CuInS_2_/ZnS in accordance with the PL spectra observed for CuInS_2_/ZnS QDs in paper [46]. The broad PL emissions are typically observed in chalcopyrite semiconductor nanocrystals, while the broad emission over 684 nm is in accordance with the one published by Li et al. [38] corresponding to CuInS_2_. The radiative recombination of excited electron-hole pairs in such nanocrystals is associated with deep defect states inside the band gap, being referred to as donor–acceptor pair recombination [6].

### 2.4. Surface Properties

**Figure 6:**
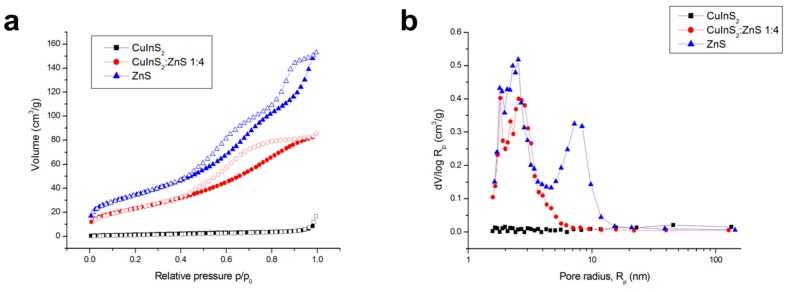
Surface properties of the synthesized nanocrystals: (**a**) adsorption-desorption isotherms and (**b**) pore size distributions.

To investigate the surface properties of the mechanochemically synthesized CuInS_2_/ZnS nanocrystals in more detail, the whole adsorption-desorption isotherms for the samples were recorded (Figure 6). The surface properties were investigated by the nitrogen adsorption method. Firstly, the S_BET_ values were determined (Table 1). It can be seen that the sample without ZnS possesses relatively small specific surface area 6 m^2^/g. This value is similar to those reported in references [39,40], in which also mechanochemical approach was used. In the case of high-temperature synthesis of CuInS_2_ starting from compounds, yielding the S_BET_ value of 30 m^2^/g was registered by Akaki et al. [47]. The addition of ZnS into the system significantly increased the S_BET_ value up to 86 m^2^/g, which is in accordance with the porous character of ZnS. The S_BET_ value of pure ZnS was determined as 125 m^2^/g, which is in accordance with the value reported by Bujňáková et al. [30]. In the mentioned paper, ZnS was also introduced by milling to different sulphide species (realgar, As_4_S_4_) and the obvious increase in S_BET_ was subsequently observed. The values of pore volume also increased after the addition of ZnS (Table 1). Regarding the average pore radius characteristics, the calculated size of the pores decreased upon the addition of ZnS, however, on the contrary to the mentioned study, this characteristic parameter was not the lowest for pure ZnS, but in the composite sample. More light on this is shed in the following part.

Further results regarding the surface properties were obtained by investigating the whole adsorption-desorption isotherms (Figure 6a). It can be seen that for the CuInS_2_ sample, the values of the adsorbed nitrogen are relatively low and the sample is almost non-porous, as there is no difference between the adsorption and desorption curve and the shape of the isotherm in the area of relative pressures around 1 indicates only a very small amount of macropores. After the addition of ZnS, the values of adsorbed nitrogen are significantly higher and also the hysteresis loop between the adsorption and desorption curve occurs, which confirms the presence of mesopores. The shape of the isotherm of pure ZnS is a little bit different from the one reported previous study [30], as seen in the present case the hysteresis loop is divided into two regions. The upper part of the loop is in accordance with the findings reported previously [30]; however, the lower part was not that large in the previous case.

The pore size distribution curves presented in Figure 6b confirm almost non-porous character of pure CuInS_2_, as very low y-axis values were registered for this sample, in comparison with the ZnS-containing ones. The addition of ZnS resulted in the mesoporous structure, as can be evidenced mainly by the broad maximum located at 2.6 nm. Almost all pores exhibit radius smaller than 7 nm. In the case of pure ZnS, the broad maximum at 7.5 nm is registered. This result is in accordance with previous study [30] and corresponds with the upper part of the hysteresis loop in the isotherm of this sample. The second maximum located at 2.5 nm was not registered previously, and this is connected with the bottom part of the isotherm. When pure ZnS and CuInS_2_/ZnS nanocomposite are compared, it can be seen that the latter sample exhibits smaller pores. Both ZnS-containing samples exhibit a small maximum under 2 nm, which is connected with the tensile strength effect [48] and is detected only when the desorption curve is used for calculations. As these maxima were not present when using adsorption curve (not shown here), they are considered artifacts.

**Figure 7:**
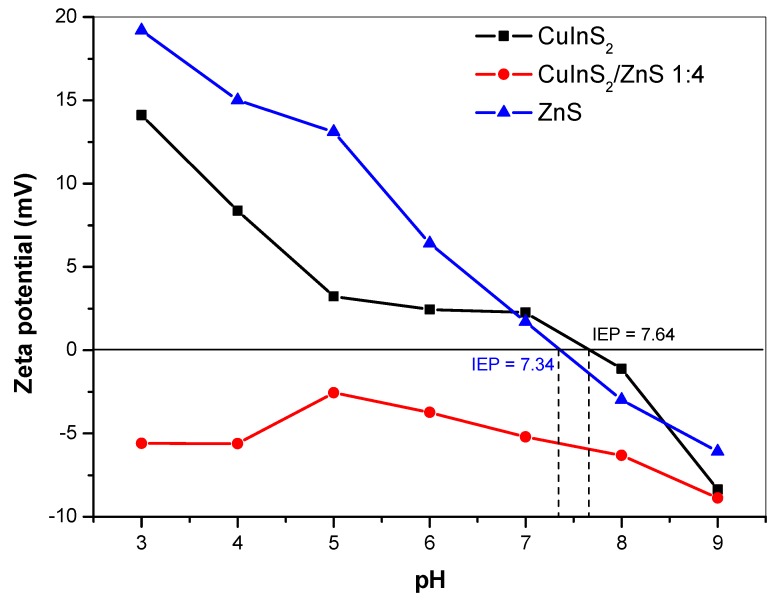
Zeta potential vs. pH of CuInS_2_, CuInS_2_/ZnS and ZnS nanocrystals measured in water.

The zeta potential (ZP) in dependence of applied pH (in a range from 3 to 9) was measured for CuInS_2_, CuInS_2_/ZnS and ZnS samples. The ZP of the samples was measured in distilled water. The results are depicted in Figure 7. In the case of CuInS_2_ sample, the isoelectric point (IEP) was detected at pH = 7.64. With decreasing pH, the particles acquired positive values (up to +14 mV at pH 3) and with increasing pH, the charge became more negative (up to −8 mV at pH 9). As described earlier [40], CuInS_2_ compound prepared by mechanochemical route had a chalcopyrite crystal structure. In this structure, each S anion is tetrahedrally coordinated to two Cu cations and two In cations [49]. The positive ZP values below the IEP are the consequence of Cu(I) and In(III) cations contribution at a crystal surface. On the other hand, the negative ZP values above IEP are a sign of sulphate and hydroxides formation.

In the case of ZnS particles dispersed in distilled water, it can be evidenced that the sample reached also positive values of ZP in almost all the entire studied pH range. The highest value of ZP was detected at pH 3 (+19 mV). With increasing pH, the ZP reached less positive values and the IEP of ZnS nanoparticles was determined at pH 7.34. Our value is considerably higher in comparison with the literature sources, where the IEPs were reported below 3.0 [50], or in the case of natural sphalerite (ZnS) at 3.0 [51], or for synthetic ZnS [52], the values in a range 3.0–3.5 were obtained. As was described earlier by Bujňáková et al. [31] and also as indicated by XRD results in this work, both the phases, sphalerite and wurtzite, were determined in ZnS prepared by mechanochemical synthesis. As was also mentioned in paper published by Liu at al. [50], the different crystal structure of wurtzite and sphalerite causes the difference in their electrokinetic behaviors. Synthetic ZnS, which was identified as wurtzite by the X-ray diffraction technique, had an IEP of 8.5, while sphalerite, another ZnS, had an IEP of less than 3.0. In summary, the obtained positive values are due to the positive Zn(II) ions present at the surface of the crystals (predominantly from wurtzite structure) and their subsequent transfer into the water. The increase is also connected with the high specific surface area (125 m^2^/g) of the mechanochemically prepared ZnS and subsequently higher amount of active sites, which are available for the dissolution of Zn(II) ions from the surface. Moreover, during the mechanochemical synthesis, a lot of defects, cracks, open pores, and intergranular spaces are created at the surface of the samples as evidenced by HRTEM in this work (Figure 4) or, e.g., in [53], and in many cases, such samples exhibit increased reactivity [24,54,55].

In a case of CuInS_2_/ZnS nanocrystals, all the ZP values lie in the negative region and the IEP was not detected in measured pH range. As described in the Materials and Methods of this paper, CuInS_2_/ZnS nanocrystals were prepared by co-milling of CuInS_2_ with precursors of ZnS. In this case, three possible scenarios could happen—surface reconstruction, interdiffusion of Zn atoms or cation exchange in the surface of CuInS_2_ [44]. Thus, the excess of sulphur ions on the surface of prepared nanocrystals could occur, which resulted in a negative charge. The confirmation of partial cation exchange between Zn and cations from CuInS_2_ (predominantly) with in [44] resulted in a blue emission shift as shown in Figure 5.

## 3. Materials and Methods

### 3.1. Mechanochemical Synthesis of CuInS_2_/ZnS Nanocrystals

**Figure 8:**
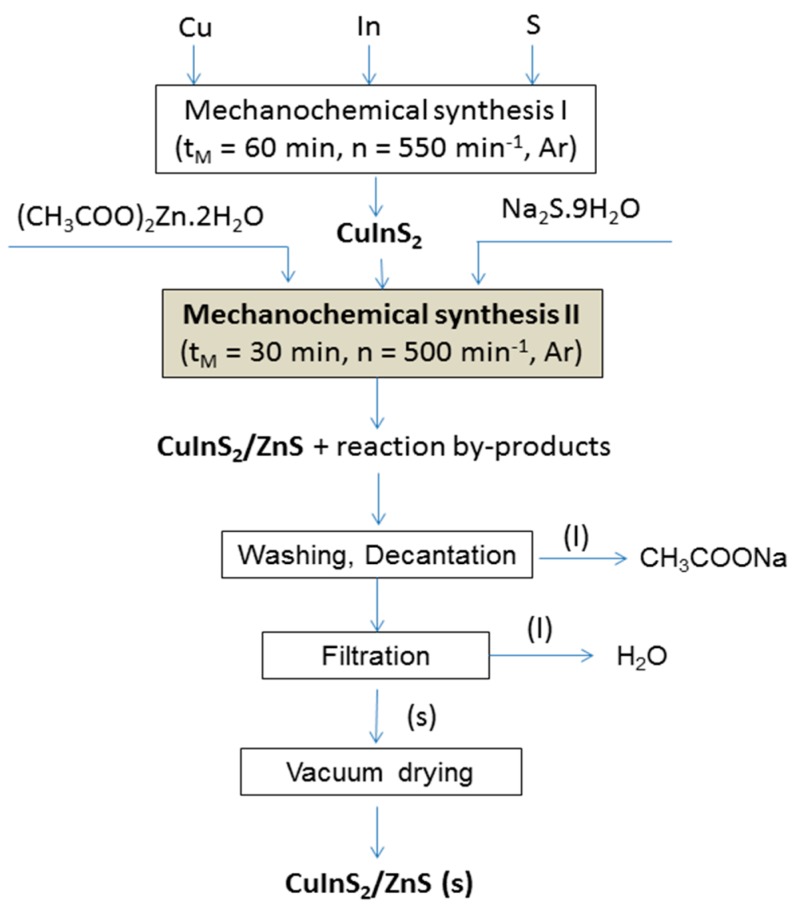
Flowsheet of the CuInS_2_/ZnS nanocrystals preparation.

CuInS_2_/ZnS nanocrystals(in molar ratio 1:4 chosen on the basis of results previously published in papers [28,29] for similar nanocrystals) were prepared by co-milling of CuInS_2_, which was prepared by milling elemental copper (99.7%, Aldrich, Germany), indium (99.99%, Aldrich, Germany) and sulphur (99%, Ites, Slovakia) according to the procedure described in previous study [40] and precursors for preparation of ZnS according to the procedures described in papers [56,57]. Precursors for ZnS were zinc acetate dihydrate (99%, Ites, Slovakia) and sodium sulphide nonahydrate (98%, Acros Organics, USA). Preparation of the CuInS_2_/ZnS nanocrystals is depicted in Figure 8 and can be described by the following reactions:

Cu + In + 2S→CuInS_2_ (1)

CuInS_2_ + (CH_3_COO)_2_Zn·2H_2_O + Na_2_S·9H_2_O→CuInS_2_/ZnS + 2CH_3_COONa + 11H_2_O (2)

Co-milling was performed in a planetary mill Pulverisette 6 (Fritsch, Germany) in an argon atmosphere for 30 min. A 250 mL tungsten carbide milling chamber with 50 tungsten carbide balls, 10 mm in diameter was used. Rotational speed of the planet carrier was 500 rpm. After the synthesis, the side product of reaction (2), sodium acetate, was removed by washing with distilled water. After vacuum drying (70 °C, 180 min), a solid phase of CuInS_2_/ZnS nanocomposite was obtained.

### 3.2. Characterization Methods

X-ray diffraction (XRD) measurements were carried out using a D8 Advance diffractometer (Bruker, Germany) equipped with a goniometer, Cu*K*_α_ radiation (40 kV, 40 mA), a secondary graphite monochromator, and a scintillation detector. All samples were scanned from 10° to 70° with steps 0.03° and 12 s counting time. Diffrac^plus^ Eva software was used for phase analysis according to the ICDD–PDF2 database.

The Raman measurements were performed in air at room temperature, with the focus of the beam of an Ar laser (514 nm) via a confocal Raman Microscope (Spectroscopy&Imaging, Warstein, Germany) in backscattering geometry. The frequency of the Raman line of crystalline Si at 520 cm^−1^ was used to calibrate the system in the present study.

Transmission electron microscopy (TEM) was used for characterization of the samples at the nanoscale. A small amount of the sample was ultrasonically homogenized in absolute ethanol for 5 min. Then, a droplet of the suspension was applied onto a lacey carbon-coated nickel grid and dried. Prior to the TEM analyses, the samples were carbon-coated to prevent charging under the electron beam. TEM analyses were performed using a 200 kV microscope JEM 2100 (JEOL, Tokio, Japan) with LaB_6_ electron source and equipped with energy dispersive X-ray spectrometer (EDS) for chemical analyses.

The adsorption isotherms and pore size distribution were obtained using a NOVA 1200e Surface Area & Pore Size Analyzer (Quantachrome Instruments, Hook, UK). The specific surface area values and pore size distribution were calculated by applying the Brunnauer-Emmet-Teller (BET) and the Barret-Joyner-Halenda (BJH) methods, respectively.

The zeta-potential (ZP) was measured using a Zetasizer Nano ZS (Malvern, UK). The Zetasizer Nano ZS measures the electrophoretic mobility of the particles, which is converted to the zeta potential by using the Helmholtz–Smoluchowski equation built in the Malvern zetasizer software. The zeta potential measurements of the samples diluted in distilled water were repeated 3 times with at least 12 subruns for each sample.

The micro-photoluminescence (micro-PL) spectra were measured by a UV-Vis-NIR confocal Raman Microscope (Spectroscopy&Imaging, Warstein, Germany). The excitation at 514 nm was carried out using an Ar laser. For measuring the PL intensity, the sample was dispersed on SiO_2_/Si substrate.

## 4. Conclusions

The CuInS_2_/ZnS nanocrystals have been synthesized in a two-step mechanochemical reaction. XRD patterns proved the coexistence of both components. The obtained CuInS_2_/ZnS nanocrystals exhibit crystallite size 8 ± 2 nm for CuInS_2_ and 2.5 ± 0.5 nm for ZnS phases. According to TEM observations, the sample is composed of agglomerated CuInS_2_ and sphalerite ZnS nanocrystallites. HRTEM analyses revealed the presence of numerous hexagonal wurtzite-type planar defects (twins and stacking faults) inside the cubic ZnS matrix, which decrease the effective crystallite size of the ZnS phase. The observed shifts in Raman and photoluminescence spectra with respect to literature data are attributed to twins and stacking faults generated during the high-energy milling. Nitrogen adsorption has shown that the addition of ZnS results in a significant enrichment of pore properties and creation of mesopores. ZP of the CuInS_2_/ZnS nanocomposite was negative in all measured pH range (3–9), which indicates excess of sulphur ions on the surface due to milling.

From the results follows that the prepared CuInS_2_/ZnS material is not just simply a mixture of the two components, rather a structural changes in the lattices/spaces inside the structure took place as a result of the milling. The both phases (larger CuInS_2_ and very small ZnS particles) were found by TEM separately. However, the results of XRD, PL, ZP, surface area and EDS measurements proved that the mutual interaction between them occurred at grain boundary, therefore the microstructure changes were observed.

The prepared CuInS_2_/ZnS nanocrystals could serve as labeling medium because of their extraordinary properties. However, for using such inorganic nanocrystals in biomedicine, it is necessary to cover them by biocompatible organic material. In the near future, its potential utilization for bioimaging applications will be studied.

## Figures and Tables

**Table 1:** Values of specific surface area, pore volume and average pore radii of the synthesized nanocrystals.

Sample	S_BET_ (m^2^/g)	Pore Volume (cm^3^/g)	Average Pore Radius (Å)
CuInS_2_	6	2.599 × 10^−2^	8.40782 × 10^1^
CuInS_2_:ZnS 1:4	86	1.32 × 10^−1^	3.06475 × 10^1^
ZnS	125	2.365 × 10^−1^	3.79239 × 10^1^
